# Some Observations Respecting the Formation of Stones in the Bladder

**Published:** 1819-12

**Authors:** John P. Baldy

**Affiliations:** Surgeon to the Storehouse Dispensary, Plymouth-Dock.


FOR THE LONDON MEDICAL AND PHYSICAL JOURNAL.

### *Some Observations respecting the Formation of Stones in the Bladder.*

By JOHN P. BALDY, Esq. Surgeon to the Storehouse Dispensary, Plymouth-Dock.

I FEEL some degree of reluctance in wishing to fill even a page of your Journal with a subject so well understood as that of the formation of stones in the bladder; but, when cases of calculi present any unusual or very remarkable circumstances, they certainly merit being recorded.

The case which I transmit to you for insertion in your Journal, has nothing in it to arrest attention, except that the nucleus on which the calculous concretions had formed themselves was a common pin, of a large kind, being full an inch and a quarter in length, and with a proportioned head. The patient, whose name is James Mountjoy, a mason by trade, had no consciousness of having at any time swallowed a pin, or that one had entered into any part of his body. He was a young man of about nineteen years of age, and healthy, excepting in this instance of disease. He had laboured under symptoms of stone in the bladder, attended with a constant diarrhœa, about twelve months before he applied to me. From his history of the disease I had but little doubt of the formation of calculus; and, on examination with the sound, I found my opinion confirmed. The patient underwent the operation very well; it was not attended with any unfavourable occurrence: and, in three weeks, he returned to his labour in perfect health.

*Plymouth-Dock; 1st October, 1819.*

